# An unusual cause of anemia in cirrhosis: spur cell anemia, a case report with review of literature 

**Published:** 2016

**Authors:** Graziella Privitera, Giovanni Meli

**Affiliations:** *Department of Clinical and Experimental Medicine, Garibaldi Hospital, University of Catania, Italy*

**Keywords:** Haemolysis, Acanthocytes, Spur cell anemia, Alcoholic liver cirrhosis, Liver transplantation

## Abstract

Chronic anemia is common in liver cirrhosis. In this setting, the pathogenesis of anemia is complex and multifactorial. Spur cell anemia is a serious disorder in cirrhotic patients and is associated with poor prognosis. Liver transplantation constitutes the only therapeutic tool. We report a case with severe spur cell anemia in alcoholic liver cirrhosis. In the attempt to investigate the origin of the disorder, we have evaluated the lipoprotein profile and found a significant reduction of apolipoprotein AI and HDL_3 _subclass as a possible cause of the disease.

## Introduction

 Cirrhosis is often associated with chronic anemia. Up to 70% of cirrhotic patients have reduced hemoglobin levels. The pathogenesis of anemia in cirrhosis is complex and multifactorial, and includes portal hypertension- induced sequestration, alterations in erythropoietin, bone marrow suppression and increased blood loss (eg. Hemorrhage, hemolysis) (1,2). We present a case with a severe anemia in alcohol- related liver cirrhosis. Anemia in alcoholic liver disease has a broad spectrum of differential diagnosis. Possible causes range from malnutrition, iron, folate, or vitamin B12 deficiency, bone marrow suppression, and even splenic sequestration (3). Severe hemolytic anemia is a rare phenomenon in patients with liver cirrhosis and could be associated with the presence of spur cells. Spur cell anemia (SCA) is a serious disorder in cirrhotic patients, indicating poor prognosis. The diagnosis should be suspected when a severe anemia requiring frequent red blood cells (RBC) transfusions is combined with progressive liver failure, jaundice, coagulopathy, and encephalopathy. Rapid resolution of SCA has been observed after liver transplantation (LT), therefore early diagnosis is crucial (4). 

## Case Report

A 44- year- old man was admitted to our hospital due to severe fatigue and jaundice started 2 weeks ago. He had a history of alcoholic cirrhosis complicated with ascites and portal hypertensive gastropathy. He referred a recent history of severe anemia that required multiple blood transfusions fortnightly without any sign of bleeding of the gastrointestinal tract. He was a hard smoker (pack/years 50) with related pulmonary emphysema.

On examination, blood pressure was 130/70 mmHg; pulse rate was 92/min; respiratory rate was 22/min; and body weight was 69.5 Kg (height 173 cm, BMI: 23.2). Physical examination revealed marked icterus of all mucous membranes and skin. The liver was felt two fingerbreadths below the right costal margin; the tip of the spleen was palpable, there was ascites and peripheral pitting edema. 

Laboratory tests at admission are shown in Table 1. The patient exhibited a severe macrocytic anemia (Hemoglobin 7.4 g/dL, Mean corpuscular volume 128 fl) with features consistent with hemolysis (haptoglobin 2 mg/dL, unconjugated bilirubin 11.2 mg/dl, Lactate dehydrogenase 581 mg/dL, and reticulocyte count 6.7% with reticulocyte index 2.97). As expected, there was an impairment of liver function tests (albumin 2.8 g/dL, total bilirubin 18.2 mg/dl, PT 29%). Iron, folic acid, B12 vitamin, kidney function, C- reactive protein, and erythrocyte sedimentation rate, were in the reference range.

**Figure 1:**
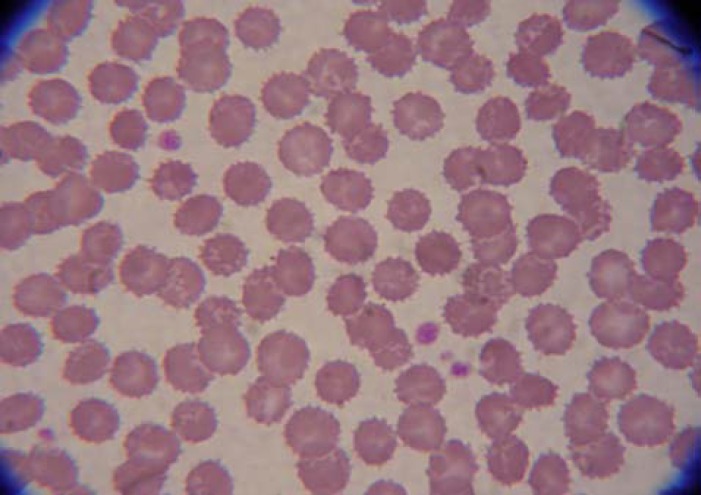
Peripheral blood smear

We diagnosed haemolytic anaemia in a patient with an advanced alcoholic liver disease (Child Pugh C, score 11). To identify the cause of haemolysis, we performed direct and indirect Coombs tests, paroxysmal nocturnal hemoglobinuria test, haemoglobin phoresis, and blood cultures, all of them resulted negative. Indeed, we evaluated anti-nuclear antibodies and procalcitonin levels, showed negative results. We also performed a peripheral blood smear, which showed a high number of acanthocytes and spur cells (90%) (Figure 1). 

To investigate the origin of SCA, apolipoprotein A1 and HDL subclasses measurement was performed. The patient showed a significant reduction of ApoA1 and HDL3 levels, in contrast HDL2 value was increased (Table 2). 

According to the severity of cirrhosis and the development of this complication, we referred the patient to a centre for orthotopic liver transplantation. The patient is currently on a waiting list for liver transplantation. 

**Assessment of Lipid fractions**

HDL subfractions were evaluated in whole plasma by the gradient gel electrophoresis, followed by Western blotting. Serum Apo-AI levels were assayed by immune-nephelometry using a commercial kit (Roche Diagnostic, Besel, Switzerland) in an automated nephelometer (RADIM, Rome, Italy). An amount of serum containing 190 ng of Apo-AI was loaded on a 4-25% acrylamide gel. Protein bands were electrotransferred to a polyvinylidene difluoride membrane (Immobilon-P, Millipore, USA), and apo-AI was revealed by a polyclonal anti-Apo-AI antibody followed by a chemiluminescent HRP- linked anti-rabbit secondary antibody (Pierce, USA). Chemiluminescent bands were quantified by Clarity image software (LSC, Lighting Systems, Australia). 

The relative positions of HDL2 and HDL3 were evaluated against fluorescently labeled high molecular weight protein markers (Amerscham GE Healthcare). The amounts of HDL2 and HDL3 were expressed as the percent of the total Apo-AI luminescence in the HDL dimension ranged between 17 and 7 nm in diameter (5).

**Table 1:** Laboratory values at admission

Blood Count (normal values)	
Leukocytes (4.3-10.3 x 10^3^ μl)	6.3
Erythrocytes (4.38-5.77 x 10^6^ μl)	1.8
Haemoglobin (13.6-17.2 g/dl)	7.4
Haematocrit (39.5-50.3%)	20
Thrombocytes (156-373 x 10^3 μl)	77
MCV (80.7-95.5 fl)	128
MHC (27.2-35.6 pg)	35.7
Differential blood count	
Neutrophils (41.0-73.0 %)	45.1
Lymphocytes (19.4-44.9 %)	37.7
Monocytes (5.1-10.9 %)	8.9
Eosinophils (0.9-6.0 %)	7.3
Basophils (0.3-1.5 %)	1
Liver/ Heart/Muscle	
CK (29.0-200.0 UI/L)	259
LDH (125-243 UI/L)	581
GOT/ AST (5-34 UI/L)	55
GPT/ ALT 0-55 (UI/L)	26
GGT (9-64 UI/L)	14
AP (56-146 UI/L)	123
Bilirubin total (0.201.20 mg/dl)	18.2
Bilirubin dir. (0.00-0.50 mg/dl)	7
Bilirubin ind. (0.00-0.50 mg/dl)	11.2
Albumin (3.5-5.0 g/dl)	2.8
Coagulation	
INR (0.90-1.10)	2.45
PT (80-120 %)	29
PTT (25-35 sec)	52
Kidney	
Urea (15-56 mg/dl)	13
Creatinine (0.60-1.30 mg/dl)	0.72
Sodium (136-145 meq/l)	136
Potassium (3.5-5.1 meq/l	3.2
Inflammatory markers	
ESR 1 h/2h (0-14 mm)	8
RCP (< 0.5 mg/dl)	0.32
Haptoglobin (40-268 mg/dl)	2
Iron ( 65-175 microg/dl)	124
Ferritin (24-336 ng/ml)	382
B12 vitamin (180-914 pg/ml)	1039
Folic acid (3-20 ng/ml)	14.3

## Discussion

Due to spur cells or acanthocytes, hemolytic anemia is an acquired disorder. Patients usually not respond to blood transfusions, since infused erythrocytes quickly acquire the “spiky” shape of spur cells. Spur cells are large erythrocytes with “spike-like” projections deforming their shape and flexibility. These morphological alterations make them susceptible to trapping and destruction by the spleen with consequent short survival and the development of anemia (6). 

**Table 2:** Lipoprotein parameters

	Normal Values	
Total Cholesterol (mg/dl)	110-200	124
HDL- Cholesterol (mg/dl)	45-110	21
LDL-Cholesterol (mg/dl)	< 160	94
Triglycerides (mg/dl)	50-150	45
Apolipoprotein A_1 _(mg/dl)	115-220	55.6
HDL_2_		0.77
HDL_3_		0.23

The pathogenesis of spur cell anemia involves changes in serum lipids that affect the lipid composition and fluidity of erythrocyte membranes in terms of cholesterol to protein and cholesterol to phospholipid ratios. In patients with severe liver disease, there is an imbalance in lipid metabolism resulting in accumulation of cholesterol in RBC membranes (7). Cicognani, et al. documented that low-density lipoprotein cholesterol (LDL-C), high-density lipoprotein cholesterol (HDL-C) and total cholesterol (TC) levels are progressively lower as liver function deteriorates (8). According to these data, we have recently shown that TC, HDL-C, LDL-C and triglycerides were significantly reduced in cirrhotic patients compared with controls and progressively decreased with worsening severity of liver disease (5). To further confirm the crucial role of lipoprotein impairment in spur cell anemia, Duhamel, et al. reported that apolipoprotein A-II levels were significantly lower in cirrhotic patients with SCA than in cirrhotics without spur cells, suggesting profound perturbations of the structure and metabolism of HDL. The reduction of Apo-AII concentration give rise to an elevation in HDL2 subfraction and a reduction of HDL3 (9). According to the importance of lipid subfractions in the pathogenesis of SCA, we measured Apolipoprotein A1 and HDL subclasses. As shown in table 2, our patient showed low levels of apoA1 and HDL3 and a high concentration of HDL2, confirming the role of lipid impairment in the process of spur cell formation.

Spur cell anemia is an insufficiently investigated entity and its clinical importance has not been documented yet. There are few reports on its prevalence and survival. Furthermore, the prevalence varies according to the different criteria used to diagnose the disease. Sousa, et al. examined 339 patients with cirrhosis and found a prevalence of SCA of 4, 13%. The diagnostic criteria used were: Hb < 10 g/dl, hemolysis, spur cells >5% in the peripheral blood, and the exclusion of other causes of anemia. All the patients were in stage C of Child- Pugh and the presence of spur cells were associated with a poor prognosis with a survival of 1 month without LT (10). Vassiliadis reported an incidence of SCA of 16.7% in 54 cirrhotic patients studied. Patients with at least 5% of spur cells, have more advanced liver disease compared with patients with less than 5% of spur cells.Affected patients showed lower three months survival rate (4). In a recent study of Alexopoulou, et al., the prevalence of SCA was evaluated in 116 cirrhotic patients. The diagnosis was based on the same criteria used in the study of Sousa, et al. The presence of spur cells was reported in 31% of patients and was strictly associated with mortality (11). Several investigators have reported the association between alcoholism and spur cell anemia, but recent reports demonstrated that the entity might be present in liver disease regardless of etiology. It was reported that the comparison of spur cell rate in patients with alcoholic cirrhosis versus the remaining etiologies did not exhibit any difference (4). As mentioned below, the presence of spur cell anemia in cirrhosis has been associated with advanced disease and poor prognosis, with a strict association with mortality. The majority of patients die within months of diagnosis. The median survival observed in the study of Sousa was 3529 days, Vassiliadis reported a survival time of 25 days and recently Alexopoulou reported a median survival of 1.9 months in patients with spur cells > 5%. In contrast, patients with evidence of spur cells of 1-4% without signs of hemolysis, did not report an increased mortality rate compared with cirrhotic patients with no evidence of spur cells (12). Therefore, the presence of spur cell rate higher or equal to 5% is a strong independent predictor of mortality and merits further investigation as a prognostic indicator in patients awaiting liver transplantation. Another crucial point is represented by the reversibility of spur cell anemia after liver transplantation. Complete resolution of SCA has been reported after LT in all the studies and case reports (12). This phenomenon might be explained to the normalization of lipid metabolism or to a decrease in portal hypertension and hypersplenism following LT. Chitale, et al. reported a case of a patient with alcoholic decompensated cirrhosis and spur cell anemia. In the patient described, the placement of transjugular intrahepatic portosystemic shunt did not resolve SCA. In contrast, a spontaneous resolution was observed after liver transplantation, suggesting that the decrease of portal pressure did not represent the pathogenetic explanation of the resolution of SCA following LT (13). Although the only effective treatment of SCA is liver transplantation, other pharmacological interventions were proposed. Aihara, et al. reported a case of successful treatment of SCA with a combination therapy consisting of flunarizine, pentoxifylline, and cholestyramine. Although limited, these observations suggest that a non-invasive therapy could be taken into consideration to decrease transfusions and hospitalization until LT is performed (14). 

The real prevalence and clinical relevance of SCA in liver cirrhosis need to be better defined, however, clinicians should be aware that SCA represents one of the causes of anemia in cirrhosis. In this setting, the evaluation of red blood cell morphology could represent a critical step in the assessment of cirrhotic patients with anemia. It can provide key information to create the differential diagnosis and to early identify patients with SCA to set up them into a higher priority for LT and improve peri- and post-LT survival rates.
